# Novel integrative genomics strategies to identify genes for complex traits

**DOI:** 10.1111/j.1365-2052.2006.01473.x

**Published:** 2006-08

**Authors:** E E Schadt

**Affiliations:** Rosetta Inpharmatics, LLC, a wholly owned subsidiary of Merck & Co., Inc. Seattle, WA 98109, USA

**Keywords:** eQTL, gene expression, gene networks, genetics, integrative genomics

## Abstract

Forward genetics is a common approach to dissecting complex traits like common human diseases. The ultimate aim of this approach was the identification of genes that are causal for disease or other phenotypes of interest. However, the forward genetics approach is by definition restricted to the identification of genes that have incurred mutations over the course of evolution or that incurred mutations as a result of chemical mutagenesis, and that as a result lead to disease or to variations in other phenotypes of interest. Genes that harbour no such mutations, but that play key roles in parts of the biological network that lead to disease, are systematically missed by this class of approaches. Recently, a class of novel integrative genomics approaches has been devised to elucidate the complexity of common human diseases by intersecting genotypic, molecular profiling, and clinical data in segregating populations. These novel approaches take a more holistic view of biological systems and leverage the vast network of gene–gene interactions, in combination with DNA variation data, to establish causal relationships among molecular profiling traits and Fbetween molecular profiling and disease (or other classic phenotypes). A number of novel genes for disease phenotypes have been identified as a result of these approaches, highlighting the utility of integrating orthogonal sources of data to get at the underlying causes of disease.

Treating DNA variations as markers and testing such markers in human and experimental populations for co-segregation with disease or other phenotypes of interest, is an approach to elucidating complex traits originally proposed by Botstein *et al.* (1980), which is in widespread use today. Markers that co-segregate with a disease trait highlight regions in the genome that are linked to and partially explain disease susceptibility. Once a genomic region has been linked to disease, positional cloning strategies and direct testing of positional candidate genes are employed to identify the gene or genes of interest predisposing to disease in the region of interest. Therefore, the primary focus of this approach is more or less restricted to looking in a single genome location for a single gene driving a disease trait of interest, a classic reductionist approach to elucidating complex systems.

While the identification of genes in a region found to be linked to disease is of high interest, a more general view is that of genetic loci associated with disease as perturbations to a biological system that ultimately result in altering the genomic, transcriptional, proteomic, and/or signalling networks in a way that increases susceptibility to disease. The set of genetic perturbations represented in any given system provide causal anchors that can aid in establishing causal associations among genes in the biological network, beyond what can be achieved by correlation data alone. While the set of genetic perturbations can change from population to population, different constellations of perturbations can affect common or completely distinct pathways that in turn lead to susceptibility to a common disease. Viewed in this way, what becomes most important in elucidating disease traits is not necessarily the identification of the genes that give rise to genetic linkages to disease, but instead the detection of the effects such perturbations have on the various biological networks driving disease.

Considering large-scale molecular profiling traits (transcriptomic, metabolomic, proteomic and so on), together with genetic linkages/associations for these and disease traits, leads to a more holistic view of the biological system under study with respect to disease. Instead of focusing only on the discovery of a single gene at the disease susceptibility locus, the genetic loci are considered as perturbations to the biological network, potentially highlighting whole classes of genes that may serve as key drivers of disease. Until recently, considering large-scale molecular profiling traits together with genetic linkages/associations to these and disease traits was not possible given the functional genomics technologies needed to take a comprehensive look at the gene networks were lacking. However, the last 10 years has seen an explosion of large-scale, high throughput functional genomics technologies that have motivated a rapid paradigm shift towards a more systems-level view of biology (Hartwell *et al.* 1999; Schadt *et al.* 2005b). With new tools now available to look at interactions among tens of thousands of genes in different tissues and in different states, the completion of the sequencing of the genome of a plethora of organisms, and a growing computational infrastructure that enables integrated views of DNA, RNA, metabolomic, and protein interaction data to elucidate the fundamental nature of disease and living systems more generally, success in biomedical research in the future will likely demand a more comprehensive view of the complex array of interactions in biological systems. How such interactions are influenced by genetic background, infection, environmental states, lifestyle choices and social structures more generally will also be important (Zerhouni 2003; Barabasi & Oltvai 2004; Schadt *et al.* 2005b).

Several groups have recently leveraged these technologies to study the genetic architecture of gene and protein expression traits and have begun to tease apart the complex network of interactions associated with complex traits (Brem *et al.* 2002, 2005; Schadt *et al.* 2003, 2005a; Bystrykh *et al.* 2005; Chesler *et al.* 2005; Ghazalpour *et al.* 2005; Mehrabian *et al.* 2005; Zhu *et al.* 2004). Here I discuss three novel integrative genomics strategies that have been applied to mouse F2 intercross populations to identify susceptibility genes for a number of metabolic traits. The early successes achieved in these and other similar studies suggest that a more integrative genomics approach to dissecting disease traits will significantly enhance the identification of key drivers of disease beyond what could be achieved by genetics alone.

## Integrating genotypic, molecular profiling and clinical data to infer causal associations among traits

**Figure 1:**
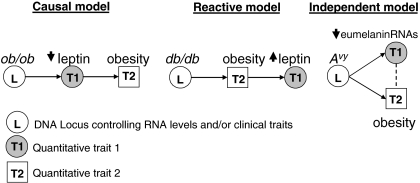
Possible simple relationships between two quantitative traits (T1 and T2) that are at least partially driven by a common genetic locus (L). Relative to T1, the Causal Model represents the simplest causal relationship of a single QTL, L, for the trait T2, where L acts on T2 through trait T1. The *ob/ob* mouse exemplifies this case, where leptin deficiency leads to obesity. The Reactive Model is the simplest reactive diagram for a single QTL, L, for the trait T2, where in this case the expression of gene T1 is responding to T2. The *db/db* mouse fits this relationship, as in this animal model leptin levels increase in response to obesity. The Independent Model results when the QTL, L, is causative for the trait T1 as well as the trait T2, but acts on these traits independently, where T1 and T2 may be correlated for reasons other than the common QTL. The A^y^ mouse provides an example of this relationship as the mutation causes decreased levels of eumelanin RNAs levels and independently gives rise to obesity.

Several groups have recently described combining genotypic data with molecular profiling data to elucidate complex traits, where one of the primary aims is the identification of molecular profiling traits and classic phenotypes that are associated with common DNA loci in a given population of interest (Brem *et al.* 2002, 2005; Schadt *et al.* 2003, 2005a; Bystrykh *et al.* 2005; Chesler *et al.* 2005; Ghazalpour *et al.* 2005; Mehrabian *et al.* 2005; Zhu *et al.* 2004). Molecular profiling traits, like many classic phenotypes, are complex, resulting from complex interactions among a number of genes and environment (Schadt *et al.* 2003, 2005a; Brem *et al.* 2005; Doss *et al.* 2005). We have also recently demonstrated that DNA variations that lead to changes in expression that in turn lead to disease susceptibility, can be distinguished in many instances from cases in which DNA variations lead to changes in disease that in turn lead to changes in expression (Schadt *et al.* 2005a). This is perhaps one of the primary utilities of leveraging genotypic and gene expression data simultaneously, given that genotypic data provides a causal anchor that can be used to enhance the ability to infer causal relationships among complex traits of interest. Towards this end, a statistical procedure to establish the relationship among traits driven by common genetic loci was recently developed (Schadt *et al.* 2005a). When two traits of interest are found to be under the control of a single genetic locus, Fig. 1 depicts the three basic relationships that are possible between the two traits. The observed correlations between any two traits in addition to the correlations between these traits and genotypes at the given locus, are the key ingredients to inferring the most likely relationship between the traits. As we have previously described (Schadt *et al.* 2005a), likelihoods for each model can be fit or conditional correlations computed to identify which of the three models in Fig. 1 best fits the data, where the best fitting model represents the most likely relationship between the traits.

**Figure 2:**
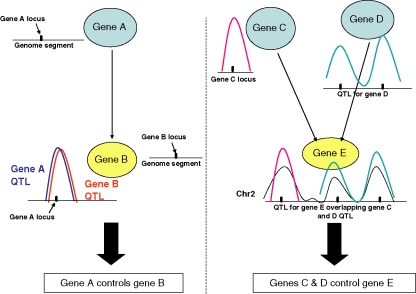
Inferring networks using molecular profiling and QTL data. The QTL data can be used to enhance the ordering of gene expression traits into simple networks. As an example, the left panel represents the simplest case where gene expression trait A gives rise to a *cis*-acting eQTL and is correlated with gene expression trait B. Gene expression trait B in turn gives rise to a *trans*-acting eQTL that is coincident with the cis eQTL for gene A. This pattern can be analysed as detailed in Fig. 1 to infer that expression variations in gene A lead to expression variations in gene B. A more complicated example is depicted in the right panel. Here gene expression trait C gives rise to a *cis* eQTL, and gene expression trait D gives rise to two *trans*-acting eQTLs. Gene expression traits C and D are also observed to be correlated with gene expression trait E. Further, gene expression trait E is also observed to give rise to three *trans* eQTL that overlap the gene C and D eQTLs. Generalizations of the arguments given in Fig. 1 can be applied to infer that this pattern suggests that expression variations in genes C and D independently lead to variations in gene E. It is of note in this example that even though gene D does not harbour mutations that directly affect is function, gene D is still identified as causal for gene E because DNA variations in other genes affect gene D's activity, which in turn affects gene E's activity. This type of reasoning can be systematically applied to all genes to reconstruct gene networks, as previously described (Zhu *et al.* 2004).

This procedure was applied to identify genes that were causal for obesity traits in an F2 intercross constructed from the B6 and DBA strains of mice (referred to here as the BXD cross) (Schadt *et al.* 2003, 2005a). A number of genes were strongly predicted as causal for this trait, and from the candidate causal gene set three genes were selected for validation via the construction of knockout and transgenic mice. All three genes were validated in this study (Schadt *et al.* 2005a). This represents one of the first applications of a more network-based view of genetics in which causal genes were identified not because their underlying genomic sequence harboured mutations that led directly to disease, but because mutations in other genes (those genes giving rise to the obesity QTL in the cross – see Fig. 2) led to changes in the expression of these three genes, and those expression changes were found to be causally associated with the obesity trait.

The procedure to order one gene relative to another using gene expression and genotypic data can be more systematically applied to hundreds or even thousands of traits to reconstruct whole gene networks (Zhu *et al.* 2004). Figure 2 illustrates how QTL information over multiple gene expression traits (or any other molecular phenotype) can be used to order a given gene expression trait with respect to other gene expression traits. Reconstructing such networks that are predictive provide a richer context within which to interpret the action of any one gene in the context of a complex disease trait (Zhu *et al.* 2004; Schadt *et al.* 2005b).

## Applying human linkage and fine mapping strategies in experimental crosses to map genes for complex traits

Leveraging experimental mouse crosses such as an F2 intercross has limitations when it comes to identifying the causal gene driving a given linkage. The above technique illustrates one approach that can get around this problem by identifying *trans*-acting expression QTL (eQTL) that overlap disease-related QTL, and by using these observations to infer causal associations. Another technique, termed ‘*in silico*’ QTL mapping, has also recently been proposed as a method to more efficiently map genes for disease (Grupe *et al.* 2001; Liao *et al.* 2004). This approach utilizes population-level disequilibrium among specific inbred lines of mice to identify loci associated with disease traits or other complex phenotypes of interest. While several studies have been published highlighting the utility of this approach in the inbred lines of mice (Grupe *et al.* 2001; Liao *et al.* 2004), several recent studies have highlighted strong linkage disequilibrium among unlinked markers operating in this population of mice (Cervino *et al.* 2005; Petkov *et al.* 2005). Nonrandom association of alleles between unlinked markers is a well-known phenomenon in human genetic studies, but only recently has been identified as a significant problem in carrying out genome-wide association studies in inbred mouse populations (Cervino *et al.* 2005). Clustered sampling, epistatic interactions among two or more loci, and population admixture are all well recognized mechanisms that can lead to this behaviour (Ott 1999), which in turn can lead to spurious genetic associations.

To address this problem and to enhance the utility more generally of the association-based studies carried out in inbred lines of mice, we proposed a combination linkage and association approach that incorporated molecular profiling data to identify genes responsible for a given linkage (Cervino *et al.* 2005). The process is similar to genetic studies commonly carried out in human populations and begins with the identification of a region in the genome linked to a disease or other phenotype of interest in an experimental cross. Identifying a region linked to disease in an experimental cross circumvents the problem of spurious association to disease that can arise because of linkage disequilibrium among unlinked loci. By leveraging haplotype information that now exists over many strains, the regions found to be linked to disease can be further narrowed by looking for shared haplotypes among strains of mice if the linkage is supported in multiple crosses (Cervino *et al.* 2005). Genetic association testing can then be carried out in the narrowed region using the population of inbred lines to identify the gene or genes that at least partially explain the specific linkage to disease. We have further enhanced this process by incorporating the type of genetics of gene expression approach discussed above. That is, if genes physically residing in the narrowed linkage region give rise to eQTL that co-localize with the physical gene location [‘*cis*’ eQTL (Schadt *et al.* 2003; Doss *et al.* 2005)], give rise to expression values that are correlated with the disease trait, and that test as causal for the disease with respect to the linkage region of interest, they are identified as primary candidate susceptibility genes for disease. This set of genes then becomes the primary set of candidate genes driving the disease.

We applied this approach to the BXD cross, a separate F2 intercross populations constructed from the B6 and C3H strains of mice (referred to here as the BXH cross) and to 62 inbred lines of mice, and mapped *Insig2* as a key cholesterol gene and as a gene associated with a number of other metabolic traits, including fat mass and insulin levels. After identifying a chromosome 1 region in the BXH cross that was linked to total plasma cholesterol levels, insulin levels and fat mass, *Insig2* was subsequently identified as a gene falling in this linkage region and giving rise to a very significant *cis* eQTL, with *Insig2* expression also supported as causal for the cholesterol trait. These observations were subsequently confirmed in a second cross (the BXD cross described herein). After identifying all SNPs in the linkage region from a panel of more than 12 000 SNPs, only three SNPs were found to associate with cholesterol levels in a broader set of inbred mice over multiple studies involving cholesterol measures. All three SNPs were in the vicinity of *Insig2*. Given the preponderance of evidence from the crosses, the expression data, the eQTL/causality tests, and the association results, we claimed *Insig2* as at least one of the susceptibility genes for cholesterol levels and other metabolic traits supporting the chromosome 1 linkage to these traits. Interestingly, after publication of our *Insig2* findings, an independent group identified human DNA variations in *Insig2* that associated with body mass index (BMI) in multiple human populations, explaining a significant proportion of lifetime BMI in the human population (Alan Herbert, personal communication, ASHG Meetings, Salt Lake City, Utah, October 2005). These human genetic results nicely confirm the connection we established in mice between *Insig2* and metabolic traits.

## Forward genetics in reverse

Another recently developed integrative genomics approach to mapping disease genes combines forward and backward genetics approaches in a way that minimizes the deficiencies of each by leveraging off of their respective strengths (Lum *et al.* 2006; Mehrabian *et al.* 2005). Like the integrative genomics approaches just described, this novel approach views QTLs as perturbation events capable of altering the transcriptional network in a given tissue, where the perturbation to the transcriptional network (the transcriptional ‘fingerprint’) is assessed by identifying those gene expression traits that link to the QTL region of interest. We proposed that the gene or genes in the QTL region of interest responsible for such a transcriptional fingerprint induced by the QTL perturbation event, could in fact be identified by perturbing candidate genes in the QTL region in independent systems and assessing the transcriptional responses. If the transcriptional fingerprint left by one of these single gene perturbation events matches the fingerprint induced by the QTL perturbation event, then we proposed that this was strong evidence that such a gene at least partially explained the QTL.

This approach was employed to identify 5-lipoxygenase (*Alox5*) as a susceptibility gene for obesity, cholesterol and bone traits in the BXD cross (Mehrabian *et al.* 2005). This followed the identification of a hot spot eQTL region on chromosome 6 in the BXD cross that was also associated with a number of clinical traits, including fat mass, bone density, and plasma cholesterol, insulin and leptin levels. Peroxisome proliferative activated receptor gamma (*Pparg*) had been considered a favoured positional candidate in this region, given that *Pparg* perturbations lead to variations in many of these same metabolic phenotypes. However, not only did *Pparg* fall in a haplotype shared by the two founding inbred strains (B6 and DBA) for the BXD cross, but the transcriptional fingerprint induced by *Pparg*-specific perturbations (rosiglitazone treatments and *Pparg* transgenic mice) did not significantly match the transcriptional fingerprint induced by the chromosome 6 QTL in the BXD cross. On the other hand, the transcriptional fingerprint for the *Alox5*-specific perturbation (*Alox5* knockout mice) did significantly match (Mehrabian *et al.* 2005). This type of procedure leverages our ability to monitor perturbations in the transcriptional network to map disease genes rapidly and efficiently. In fact, one can imagine constructing single-gene perturbation compendia in different cell types using RNAi-based technologies, and then using these compendia as a screening tool to isolate genes that may be responsible for QTL-based transcriptional perturbations in experimental crosses and beyond.

## Conclusion

Classic forward genetic approaches for dissecting complex traits are based on a reductionist approach to elucidating complex systems that are typically focused on single genes or proteins. For Mendelian traits this approach has achieved great success, but success in the common human diseases has been plagued by a great many cases in which associations have failed to replicate across human populations and QTLs have failed to reproduce across different experimental crosses, in addition to many other issues that have prevented greater success in the field. However, that phenotypically similar populations will not reproduce linkages and/or associations for a particular complex trait is perhaps to be expected, as multiple perturbations in the genetic network can affect related or completely distinct pathways that ultimately lead to the same disease. Genetic perturbations underlying disease in a given population represent just one of many ways biological networks may be perturbed in ways that lead to disease or other complex traits of interest. However, it is likely that there are many key nodes in biological networks that associate with disease but that, in fact, harbour no common DNA variations in human or other populations that predispose to common forms of disease. HMG-CoA reductase is an excellent example of a gene for which no common DNA variations have been found that associate with plasma cholesterol levels in human populations (Garcia *et al.* 2001), even though this gene is well known to play a central role in cholesterol homeostasis and is the target of statin therapies.

The integrative genomics approaches discussed herein are not limiting in this same way. Integrating genetic and molecular profiling data allows one to capture the constellation of perturbations because of variations in DNA that affect portions of the gene network that in turn lead to disease. That is, while there may only be a single gene underlying a given linkage to disease, such perturbation events may in turn cause transcript abundances of hundreds of genes to change, and many of these same genes may capture information from multiple genetic loci associated with the same disease. This in fact was the case for each of the three genes (*Tgfbr2*, *C3ar1* and *Zfp90*) we recently reported as being causal for obesity in the BXD cross (Schadt *et al.* 2005a). Combining the genetic and molecular profiling data allows for a much broader net to be cast over the set of molecular changes associated with disease, potentially providing for significantly increased power to detect causal associations to disease than could otherwise be achieved using only the genetic or molecular profiling data.

The more systematic application of causality-like arguments can be used to reconstruct whole networks (Fig. 2), where the networks provide a context in which to interpret key genes identified for disease (Zhu *et al.* 2004; Schadt *et al.* 2005b). From the standpoint of treating disease, the utility of these predictive networks includes refining the definition of disease, identifying multiple drug targets that are causal for a given disease, and prioritizing those targets based on the extent of causal association to disease, in addition to the extent of causal association with co-morbidities of the disease or with adverse events that may ensue in targeting a given pathway with pharmaceutics. Achieving this level of understanding of disease has the potential to move us away from the ‘one drug fits all’ paradigm that dominates the pharmaceutical landscape at present, and to instead deliver on the personalized medicine promise of getting the right drug to the right person at the right time.

## References

[b1] Barabasi AL, Oltvai ZN (2004). Network biology: understanding the cell's functional organization. Nature Reviews Genetics.

[b2] Botstein D, White RL, Skolnick M, Davis RW (1980). Construction of a genetic linkage map in man using restriction fragment length polymorphisms. American Journal of Human Genetics.

[b3] Brem RB, Yvert G, Clinton R, Kruglyak L (2002). Genetic dissection of transcriptional regulation in budding yeast. Science.

[b4] Brem RB, Storey JD, Whittle J, Kruglyak L (2005). Genetic interactions between polymorphisms that affect gene expression in yeast. Nature.

[b5] Bystrykh L, Weersing E, Dontje B (2005). Uncovering regulatory pathways that affect hematopoietic stem cell function using ‘genetical genomics’. Nature Genetics.

[b6] Cervino AC, Li G, Edwards S (2005). Integrating QTL and high-density SNP analyses in mice to identify Insig2 as a susceptibility gene for plasma cholesterol levels. Genomics.

[b7] Chesler EJ, Lu L, Shou S (2005). Complex trait analysis of gene expression uncovers polygenic and pleiotropic networks that modulate nervous system function. Nature Genetics.

[b8] Doss S, Schadt EE, Drake TA, Lusis AJ (2005). Cis-acting expression quantitative trait loci in mice. Genome Research.

[b9] Garcia CK, Mues G, Liao Y, Hyatt T, Patil N, Cohen JC, Hobbs HH (2001). Sequence diversity in genes of lipid metabolism. Genome Research.

[b10] Ghazalpour A, Doss S, Sheth SS, Ingram-Drake LA, Schadt EE, Lusis AJ, Drake TA (2005). Genomic analysis of metabolic pathway gene expression in mice. Genome Biology.

[b11] Grupe A, Germer S, Usuka J, Aud D, Belknap JK, Klein RF, Ahluwalia MK, Higuchi R, Peltz G (2001). In silico mapping of complex disease-related traits in mice. Science.

[b12] Hartwell LH, Hopfield JJ, Leibler S, Murray AW (1999). From molecular to modular cell biology. Nature.

[b13] Liao G, Wang J, Guo J (2004). In silico genetics: identification of a functional element regulating H2-Ealpha gene expression. Science.

[b14] Lum PY, Chen Y, Zhu J, Lamb J, Melmed S, Wang S, Drake TA, Lusis AJ, Schadt EE (2006). Elucidating the murine brain transcriptional network in a segregating mouse population to identify core functional moduels for obesity and diabetes. Journal of Neurochemistry.

[b15] Mehrabian M, Allayee H, Stockton J (2005). Integrating genotypic and expression data in a segregating mouse population to identify 5-lipoxygenase as a susceptibility gene for obesity and bone traits. Nature Genetics.

[b16] Ott J (1999). Analysis of Human Genetic Linkage.

[b17] Petkov PM, Graber JH, Churchill GA, Dipetrillo K, King BL, Paigen K (2005). Evidence of a large-scale functional organization of mammalian chromosomes. PLoS Genet.

[b18] Schadt EE, Monks SA, Drake TA (2003). Genetics of gene expression surveyed in maize, mouse and man. Nature.

[b19] Schadt EE, Lamb J, Yang X (2005a). An integrative genomics approach to infer causal associations between gene expression and disease. Nature Genetics.

[b20] Schadt EE, Sachs A, Friend S (2005b). Embracing complexity, inching closer to reality. Science's STKE: Signal Transduction Knowledge Environment.

[b21] Zerhouni E (2003). Medicine. The NIH Roadmap. Science.

[b22] Zhu J, Lum PY, Lamb J (2004). An integrative genomics approach to the reconstruction of gene networks in segregating populations. Cytogenetic and Genome Research.

